# Lovastatin improves impaired synaptic plasticity and phasic alertness in patients with neurofibromatosis type 1

**DOI:** 10.1186/1471-2377-13-131

**Published:** 2013-10-02

**Authors:** Florian Mainberger, Nikolai H Jung, Martin Zenker, Ute Wahlländer, Leonie Freudenberg, Susanne Langer, Steffen Berweck, Tobias Winkler, Andreas Straube, Florian Heinen, Sofia Granström, Victor-Felix Mautner, Karen Lidzba, Volker Mall

**Affiliations:** 1 Department of Pediatrics, Technical University Munich, Kinderzentrum München gemeinnützige GmbH, Heiglhofstrasse 63, 81377 Munich, Germany; 2 Institute of Human Genetics, University Hospital Erlangen and Institute of Human Genetics, University Hospital Magdeburg, Magdeburg, Germany; 3 Institut for General Medicine Ludwig-Maximilians-University Munich, Munich, Germany; 4 Schön Klinik Vogtareuth, Vogtareuth, Germany; 5 Department of Neurology, Ludwig-Maximilians-University Munich, Munich, Germany; 6 Department of Paediatric Neurology and Developmental Medicine, Dr von Hauner’s Children’s Hospital, Ludwig-Maximilians-University Munich, Munich, Germany; 7 Department of Neurology, University Medical Centre, Hamburg-Eppendorf, Germany; 8 Department of Neuropediatrics, University Children’s Hospital Tübingen, Tübingen, Germany; 9 Division of Neuropaediatrics and Muscular Disorders, Department of Paediatrics and Adolescent Medicine, University Hospital Freiburg, Freiburg, Germany

**Keywords:** Transcranial magnetic stimulation (TMS), Paired associative stimulation (PAS), Long-term potentiation (LTP), Synaptic plasticity, RAS-pathway, Developmental disorder, NF1, Attention, Lovastatin

## Abstract

**Background:**

Neurofibromatosis type 1 (NF1) is one of the most common genetic disorders causing learning disabilities by mutations in the neurofibromin gene, an important inhibitor of the RAS pathway. In a mouse model of NF1, a loss of function mutation of the neurofibromin gene resulted in increased gamma aminobutyric acid (GABA)-mediated inhibition which led to decreased synaptic plasticity and deficits in attentional performance. Most importantly, these defictis were normalized by lovastatin. This placebo-controlled, double blind, randomized study aimed to investigate synaptic plasticity and cognition in humans with NF1 and tried to answer the question whether potential deficits may be rescued by lovastatin.

**Methods:**

In NF1 patients (*n* = 11; 19–44 years) and healthy controls (HC; *n* = 11; 19–31 years) paired pulse transcranial magnetic stimulation (TMS) was used to study intracortical inhibition (paired pulse) and synaptic plasticity (paired associative stimulation). On behavioural level the Test of Attentional Performance (TAP) was used. To study the effect of 200 mg lovastatin for 4 days on all these parameters, a placebo-controlled, double blind, randomized trial was performed.

**Results:**

In patients with NF1, lovastatin revealed significant decrease of intracortical inhibition, significant increase of synaptic plasticity as well as significant increase of phasic alertness. Compared to HC, patients with NF1 exposed increased intracortical inhibition, impaired synaptic plasticity and deficits in phasic alertness.

**Conclusions:**

This study demonstrates, for the first time, a link between a pathological RAS pathway activity, intracortical inhibition and impaired synaptic plasticity and its rescue by lovastatin in humans. Our findings revealed mechanisms of attention disorders in humans with NF1 and support the idea of a potential clinical benefit of lovastatin as a therapeutic option.

## Background

Neurofibromatosis type 1 (NF1) is one of the most common genetic disorders occurring in 1:3.000 persons. It is caused by a loss of function mutation of the neurofibromin gene leading to rat sarcoma (RAS) pathway hyperactivity [1,2]. Beside a wide range of symptoms caused by RAS pathway, cognitive impairment is observed in about 30-65% of children with NF1 [3-5]. Furthermore, patients with NF1 show a high frequency of attention deficit disorder (Hyman 38%, Mautner 49.5%) [6,7].

It is widely accepted that synaptic plasticity plays a central role in all kinds of cognitive processes like learning and attention and, therefore, is discussed to be impaired in cognitive disorders. The pathophysiology of cognitive deficits in NF1 has been studied using a mouse model. NF1 mice show deficits in spatial learning and in attention [8]. Although distinct underlying circuits and brain mechanism mediate these different cognitive functions, it has been shown that both, memory and attention are impaired by deficits in synaptic plasticity caused by RAS pathway hyperactivity [9-11]. In interneurons, where neurofibromin expression is critical for cognitive function [4], increased RAS pathway activity causes GABA release and subsequent diminishes synaptic plasticity in mice [12]. Lovastatin, a specific inhibitor of 3-hydroxy-3-methylglutaryl coenzyme A (HMG-CoA), commonly used for the treatment of hypercholesterolemia, has been identified as potent inhibitor of RAS/mitogen-activated protein kinase (MAPK) activity. Most importantly, down regulation of the hyperactive RAS pathway by lovastatin leads to an improvement of synaptic plasticity and restores learning deficits and attention in mouse models of NF1. In humans with NF1, Krab and colleagues performed a 12-week trial with simvastatin but could not improve cognitive function in children with NF1 [13]. In contrast, an open phase 1 study revealed an improvement of verbal and non-verbal memory after three month lovastatin treatment in children [14], which could be explained by a specific function of lovastatin.

In animal models, synaptic plasticity is studied using stimulation paradigms resulting in potentiation of synaptic transmission, so called long-term potentiation (LTP). LTP can be induced by associative stimulation and is associated with learning a new motor skill [15]. Furthermore, studies using the NF1 mouse model as well as others revealed strong RAS-pathway dependency of LTP in the cell model [9,16,17].

In humans, the term LTP-like plasticity has been established [18,19]. It refers to neuronal plasticity of the human motor cortex taking place on a synaptic level which is associated to the acquisition of motor skills [19-21]. LTP is studied by transcranial magnetic stimulation (TMS) and can be induced by associative stimulation [19,22]. Furthermore, TMS can evaluate intracortical inhibition reflecting intracortical inhibition using a paired pulse paradigm with a preceding subthreshold stimulus and short interstimulus intervals (1–5 ms) [23].

In this placebo-controlled double blind study, we aimed to investigate the potential impact of lovastatin on synaptic plasticity, intracortical inhibition and attention in patients with NF1.

## Results

As attention might influence PAS effects, we controlled this in both cohorts and do not consider this as a confounding factor.

### Exp 1a: synaptic plasticity

**Figure 1:**
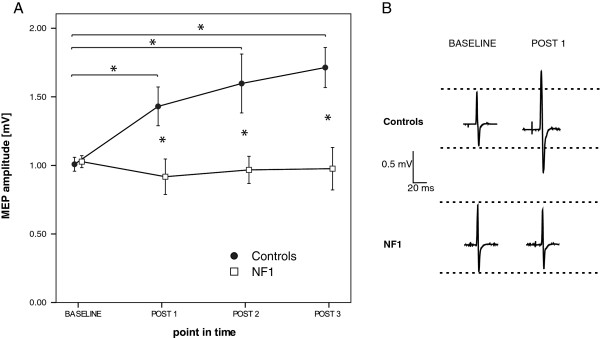
**Synaptic plasticity in patients with NF1 and controls. A)** Depicted is the time course of MEP amplitudes after PAS in healthy controls and patients with NF1. PAS led to a significant MEP increase only in the control group. Abscissa pictures point in time and ordinate mean MEP amplitude. Asterisks indicate significant differences between two groups or points in time (p < 0.05, unpaired *t* test), error bars represent ± standard error of the mean. **B)** MEP amplitudes from two representative subjects of controls and NF1before and at POST 1 after PAS. Shown are averages of 20 MEP trials in each case.

In this experiment we investigated PAS-induced LTP-like plasticity in healthy controls and patients with NF1. Motor evoked potentials increased in amplitudes in healthy controls (baseline: 1.00 ± 0.17; POST 1: 1.44 ± 0.47; POST 2: 1.60 ± 0.71; POST 3: 1.71 ± 0.48), but not in patients with NF1 after PAS (baseline: 1.03 ± 0.15; POST 1: 0.92 ± 0.43; POST 2: 0.97 ± 0.33; POST 3: 0.98 ± 0.51; see Figure 1). We performed rmANOVA on untransformed MEP data, which revealed significant main effects (TIME: *F*_[3;60]_ = 4.269, *p* = 0.011; GROUP: *F*_[1;20]_ = 10.243, *p* = 0.004), and significant interaction (*F*_[3;60]_ = 5.854, *p* = 0.002). Post hoc analysis demonstrated that the increase in MEP amplitudes was significant in the control group for all points in time (POST 1 (*p* = 0.009), POST 2 (*p* = 0.016) and POST 3 (*p* = 0.001)), but not in patients group and that there were significant differences between both groups at POST 1 (*p* = 0.014), POST 2 (*p* = 0.015) and POST 3 (*p* = 0.002). For more detail see also Figure 1.

There was no significant difference in baseline MEP amplitudes between groups (NF1: 1.03 ± 0.15 mV; healthy controls: 1.00 ± 0.17 mV; p = 0.782). Healthy controls and patients did not differ in regard to resting motor threshold (NF1: 39.55 ± 7.42%; healthy controls: 38.36 ± 6.02%; p = 0.686) or perceptual threshold (NF1: 0.54 ± 0.22 mA; controls: 0.61 ± 0.17 mA; p = 0.430). RmANOVA of resting motor threshold data revealed no significant main effects or interaction (TIME: *F*_[3;60]_ = 0.263, p = 0.844; GROUP: *F*_[1;20]_ = 0.612, p = 0.444; TIME*GROUP: *F*_[3;60]_ = 0.819, p = 0.484).

### Exp. 1b: intracortical inhibition

**Figure 2:**
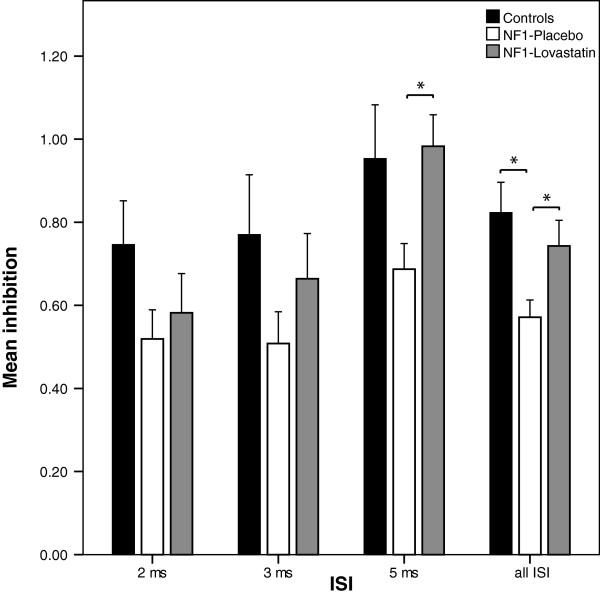
**SICI in healthy controls and patients with NF1 in on/off medication.** Depicted are data of 10 patients of NF1 and healthy controls in on/off medication. Mean inhibition at 60% resting motor threshold (for more details see methods) of the conditioning stimulus was built for ISI of 2, 3, 5 ms and over all ISI. Here, a trend towards increased inhibition in patients with NF1 (treated with placebo but not with lovastatin) compared to healthy controls can be observed. Error bars represent ± standard error of the mean.

In experiment 1b we tested intracortical inhibition in patients with NF1 and healthy controls. Patients with NF1 showed an increased SICI compared to healthy controls (ISI of 2 ms: 0.75 ± 0.33 to 0.51 ± 0.22 (*p* = 0.190), 3 ms: 0.77 ± 0.46 to 0.51 ± 0.24 (*p* = 0.190), 5 ms: 0.95 ± 0.41 to 0.69 ± 0.20, (*p* = 0.089), and over all ISI: 0.82 ± 0.40 to 0.57 ± 0.23, (*p* = 0.012; see Figure 2)).

### Exp. 1c: attention

**Figure 3:**
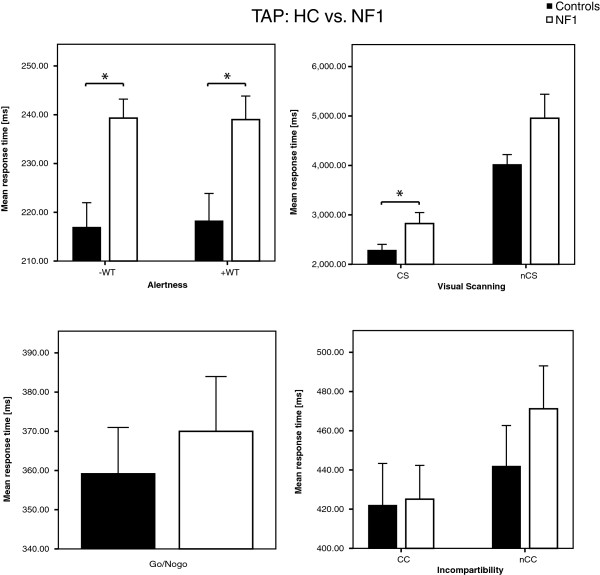
**Reaction times of TAP in healthy controls and patients with NF1.** Data of 10 patients with NF1 and healthy controls are plotted for Alertness, Go/NoGo, Visual Scanning and Incompatibility. Here patients with NF1 (treated with placebo but not with lovastatin) scored in lower ranges than healthy controls. The significance was set at a level of *p* < 0.05 and error bars represent ± standard error of the mean. Alertness: –WT (without warning tone), +WT (with warning tone); Visual Scanning: CS (critical stimulus), nCS (non critical stimulus); Incompartibility: CC (critical condition), nCC (non critical condition).

In experiment 1c attentional performance was examined in patients with NF1 and healthy controls. In most subscales of the test of attentional performance task (TAP) patients with NF1 scored in lower ranges than healthy controls (see Figure 3). Notably, deficits became manifest in the subscales alertness (-WT, +WT) and Visual Scanning (critical stimulus) where 10.33% (-WT), 9.53% (+WT) respectively 23.86% (critical stimulus). Here, the present sample scores were significantly (Alertness: -WT: 0.003; +WT: 0.012; Visual Scanning: critical stimulus: 0.047) lower than in healthy controls. In both conditions of alertness, with and without a warning tone, we observed significant faster reaction times in the healthy control group than in the patients group (without warning tone (-WT): HC: 216.9 ± 16.02 ms; NF1: 239.3 ± 12.3 ms; unpaired *t*-test: *p* = 0.003, with warning tone (+WT): HC: 218.2 ± 17.87 ms; NF1: 239 ± 15.26; unpaired *t*-test: *p* = 0.012).

### Exp2: placebo controlled double blind intervention with lovastatin

#### ***Exp 2a synaptic plasticity***

This experiment investigated PAS-induced LTP-like plasticity in patients with NF1 in an on/off-medicated condition with lovastatin.

##### Prestudy

After a single dose of 200 mg of lovastatin, there was a trend towards increased MEP amplitudes after PAS in patients with NF1 (baseline: 1.06 ± 0.11; POST 1: 1.05 ± 0.43; POST 2: 1.15 ± 0.56; POST 3: 1.25 ± 0.80) which did not reach significance (data not shown).

##### Main study

**Figure 4:**
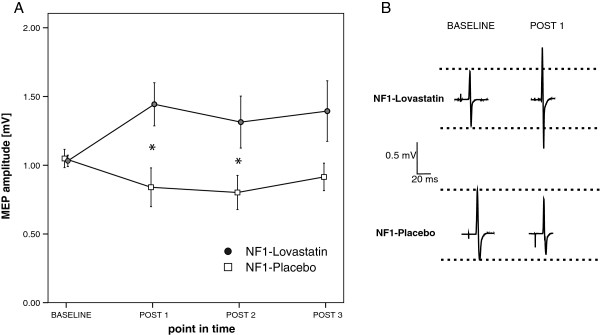
**Time course of MEP amplitudes of patients with NF1 after PAS with and without lovastatin. A)** Shown are data from the NF1 patients (*n* = 11). Abscissa depicts point in time and ordinate mean MEP amplitude. Administration of lovastatin 4 days before PAS led to a rescue of motor cortex plasticity in patients. Asterisks indicate significant differences between the two drug conditions (*p* < 0.05, paired *t* test) and error bars represent ± standard error of the mean. **B)** MEP amplitudes from one representative subject for placebo and lovastatin condition before and at POST 1 after PAS. Shown are averages of 20 MEP trials in each case.

After a four day course of lovastatin, an increase of MEP after PAS was seen but not after placebo (NF1-lovastatin: baseline: 1.03 ± 0.14, POST 1: 1.44 ± 0.52, POST 2: 1.31 ± 0.63, POST 3: 1.39 ± 0.73; NF1-placebo: baseline: 1.05 ± 0.22, POST 1: 0.84 ± 0.47, POST 2: 0.80 ± 0.41, POST 3: 0.92 ± 0.33; see Figure 4). RmANOVA on untransformed MEP data of lovastatin- and placebo-medicated patients with NF1 revealed significant main effects in DRUG (*F*_[1;20]_ = 7.730, *p* = 0.012) and DRUG × TIME interaction (*F*_[3;60]_ = 2.925, *p* = 0.047), but not in TIME alone (*F*_[3;60]_ = 0.500, *p* = 0.666). Post hoc analysis demonstrated that there was a significant difference between NF1-lovastatin and NF1-placebo in POST 1 (*p* = 0.016), POST 2 (*p* = 0.041) but not in POST 3 (*p* = 0.065).

There was no significant difference in baseline MEP amplitudes (NF1-placebo: 1.05 ± 0.22 mV; NF1-lovastatin: 1.03 ± 0.14 mV; *p* = 0.762), resting motor threshold (NF1-placebo: 41.64 ± 4.74%; NF1-lovastatin: 39.73 ± 5.64%; *p* = 0.089) or perceptual threshold (NF1-placebo: 0.71 ± 0.35 mA; NF1-lovastatin: 0.73 ± 0.26 mA; *p* = 0.749) between off and on medicated patients with NF1. RmANOVA of resting motor threshold data revealed no significant main effects or interaction (TIME: *F*_[3;60]_ = 1.720, p = 0.175; GROUP: *F*_[1;20]_ = 0.306, *p* = 0.586; TIME*GROUP: *F*_[3;60]_ = 0.785, *p* = 0.502).

#### ***Exp. 2b: intracortical inhibition***

Here we examined the influence of lovastatin and placebo on intracortical inhibition in patients with NF1. Comparing SICI after a four day course of lovastatin and after a four day course of placebo patients with NF1 did show lower SICI after lovastatin compared to placebo (inter-stimulus-intervall of 2 ms: 0.58 ± 0.30 to 0.52 ± 0.22 (*p* = 0.415); 3 ms: 0.66 ± 0.34 to 0.51 ± 0.24 (*p* = 0.221); 5 ms: 0.99 ± 0.24 to 0.69 ± 0.20 (*p* = 0.017); all: 0.74 ± 0.34 to 0.57 ± 0.23 (*p* = 0.011; see Figure 2). Furthermore differences in SICI between patients after a 4-day course of lovastatin compared to healthy controls were not seen (2 ms: 0.75 ± 0.33 to 0.58 ± 0.30 (*p* = 0.315); 3 ms: 0.77 ± 0.46 to 0.66 ± 0.34 (*p* = 0.684); 5 ms: 0.95 ± 0.41 to 0.99 ± 0.24 (*p* = 0.481); all: 0.82 ± 0.40 to 0.74 ± 0.34 (*p* = 0.734).

#### ***Exp. 2c: attention as measured with TAP in patients with NF1, intervention with lovastatin***

**Figure 5:**
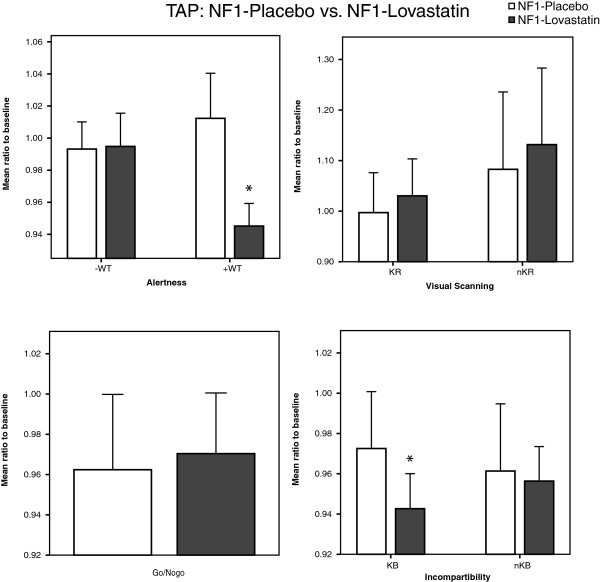
**Reaction times of TAP in patients with NF1 with placebo or lovastatin medication.** Data of 10 patients with NF1 are plotted for Alertness, Go/NoGo, Visual Scanning and Incompatibility. For phasic alertness and Incompatibility (KB) patients with NF1 (treated with lovastatin) scored in lower ranges than patients treated with placebo. The significance was set at a level of *p* < 0.05 and error bars represent ± standard error of the mean. Alertness: –WT (without warning tone), +WT (with warning tone); Visual Scanning: CS (critical stimulus), nCS (non critical stimulus); Incompartibility: CC (critical condition), nCC (non critical condition).

In experiment 2c we tested the effect of lovastatin on attentional performance in patients with NF1. Here patients with NF1 demonstrated faster reaction times after administration of the drug in the case of the condition with a warning tone but not without the warning tone (see Figure 5). In the condition with the warning tone, the reaction time was significantly shorter (from 240.5 ± 21.69 ms at baseline to 226.9 ± 18.3 ms after lovastatin administration, *p* = 0.004, (level of significance after Bonferroni-Holm adjustment: *p* ≤ 0.007), whereas placebo had no effect (baseline: 233.1 ± 13.45 ms to 235.4 ± 17.91 ms).

## Discussion

The main finding of the present placebo-controlled, randomized double blind study is that lovastatin normalizes LTP-like plasticity, decreases intracortical inhibition and improves phasic alertness in patients with NF1.

Impaired LTP-like plasticity, has also been described in neuropsychiatric conditions such as Alzheimer’s disease [24], in patients with schizophrenia [25] as well as in patients with Parkinson’s disease [26,27]. Taking into account that synaptic plasticity is regarded as central mechanism of learning and memory, impairment in neuropsychological disorders may be consequent. Results of our study revealed a RAS pathway specific impaired LTP-like plasticity in humans that was improved by a pathway specific pharmacological intervention with lovastatin in a placebo-controlled, randomized double-blind design.

Lovastatin, a specific inhibitor of 3-hydroxy-3-methylglutaryl coenzyme A (HMG-CoA), has been shown to reduce RAS-pathway hyperactivity in NF1 mice [8,28] and in consequence to restore synaptic plasticity, normalize inhibition and to improve learning disability and attention. Therefore, we performed a placebo-controlled randomized study. Indeed, a 4-day course of lovastatin normalized increased intracortical inhibition, improved LTP-like plasticity and alertness in patients with NF1, whereas placebo intervention exposed no effects. Because of the placebo-controlled randomized double blind cross over design, confounding factors such as differences in attention were best possible excluded. It supports the assumption that lovastatin as a pathway specific intervention is responsible for our finding which may elucidate the role of the RAS pathway in LTP-like plasticity.

The concept of LTP-like plasticity is based on the assumption that effects of paired associative stimulation on MEP amplitudes show comparable attitudes as associative plasticity in in vitro studies:long-lasting, input-specific, N-Methyl-D-Aspartat (NMDA)-receptor dependent, rapidly evolving, fully reversible, and bidirectional. This study contributes significantly to this concept. In vitro it has been shown that RAS-pathway activity is significantly related to LTP induction which has now been shown to be the case for LTP-like plasticity as well.

In healthy subjects, we recently demonstrated that increasing intracortical inhibition by repetitive slow frequency (0.1 Hz) TMS diminishes the induction of LTP-like plasticity [21]. On the other hand, decreasing intracortical inhibition has been shown to improve induction of synaptic plasticity [29]. These findings lead to the concept of a balance between gating and anti-gating of synaptic plasticity which is regulated by intracortical inhibition [30] and which may conclusively explain our results. First, increased intracortical inhibition in patients with NF1 compared to healthy controls may lead to a reduction of LTP-like plasticity (increased anti-gating). Second, decrease in intracortical inhibition through lovastatin may lead to an improvement of LTP-like plasticity (shift toward gating). This hypothesis is supported by our findings of a decreased intracortical inhibition through lovastatin showing a significant decrease at the interstimulus interval of 5 milliseconds and a trend of a decrease at 2 and 3 milliseconds, although the lack of significance of the latter has to be taken as a limitation.

This raises the question of the impact of lovastatin on behavioural parameters. Attention has been shown to be impaired in RAS pathway hyperactivity, to be related to intracortical inhibition on a neurophysiological basis and decreased synaptic plasticity and it has been shown to be sensitive to pharmacological interventions. This makes attention to a predestined parameter to evaluate cognition after a shot term intervention in RAS pathway disorders with lovastatin. We postulated to find impairments of behavioural parameters in our cohort of patients with increased intracortical inhibition and decreased synaptic plasticity. Indeed, we observed deficits in tonic (reaction time without WT) and phasic alertness (reaction time with WT) as well as deficits in visual scanning in patients with NF1 compared to healthy controls. Additionally, we demonstrated phasic alertness to be significantly improved after a 4-day course of lovastatin. However, the time period of pharmacological intervention was short. Phasic alertness – the ability to increase attention for a short time period on demand - may be considered as one of the most basic cognitive tasks examined in our study and, therefore, may be responsive even to a short term intervention. More complex tasks, such as tonic (continued) alertness, visual scanning, incompatibility and impulsivity may need a longer time period to respond to pharmacological intervention. Recently, in an open phase 1 study focusing on a safe dosage of lovastatin in children with NF1, an improvement of verbal and non-verbal memory after three month lovastatin treatment was observed [14].

## Conclusions

In conclusion, our study demonstrated, for the first time, that RAS-pathway specific modulation by lovastatin, given in a placebo-controlled, randomized double blind design, restores LTP-like plasticity, decreases intracortical inhibition and improves phasic alertness in patients with NF1. This study may be interpreted as a “proof of principle” study elucidating pathophysiology of cognitive impairment in NF1 and raises the question of the potential therapeutic impact of lovastatin in NF1. Our study may encourage clinical trials answering this question.

## Methods

The study was approved by the local Ethics Committee of the University Hospital of Freiburg (41/08), Germany and all subjects gave written informed consent. It was conducted according to the latest version of the Declaration of Helsinki.

### Experimental design

#### ***Experiment 1 (Exp. 1): Comparing healthy subjects and patients with NF1***

**Figure 6:**
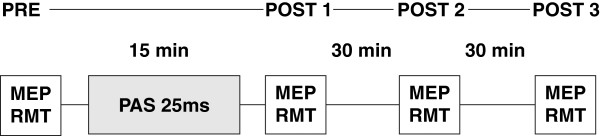
**Timeline of PAS experiment.** We measured motor evoked potentials (MEP) amplitudes as well as resting motor threshold before and at three points in time after paired associative stimulation (PAS). PAS was performed with an interstimulus interval of 25 ms.

We compared healthy subjects with patients with NF1 testing the hypothesis whether patients with NF1 (a) show impairment of synaptic plasticity as tested by the TMS paradigm of Paired associative stimulation (PAS; for experimental design see Figure 6), (b) show increased level of intracortical inhibition as tested by the TMS paradigm of short interval cortical inhibition (SICI) and (c) have an attention deficit as tested with the test for attentional performance (TAP) using the domains alertness, visual scanning, go/no go and incompatibility. In Exp 1b and 1c healthy subjects were compared to the placebo group (NF1 patients) of Exp 2b and 2c in order to reduce the testing time of each patient and minimize retesting carry-over effects.

Since experiments may interfere with each other concerning influence in attention and compliance as well as the total amount of transcranial magnetic stimuli before PAS and influence of PAS on intracortical inhibition, the subparts a, b and c in both experiments were performed separately with a time delay of at least two weeks [31].

#### ***Experiment 2 (Exp. 2): placebo controlled double blind intervention with lovastatin***

We performed a pre study using a single dose of 200 mg of lovastatin. Because we found a trend but not a significant effect on synaptic plasticity as measured by PAS (data not shown) the placebo-controlled, double blind main study was performed with 200 mg lovastatin daily for four days before the experiments. Although this dosage is significantly higher than the clinical routine dose, it was considered to be save as previously shown [32]. Indeed none of our patients reported of any side effect at all. The study was divided into three sub studies (2a-2c) using the same testing procedure as in Exp1.

### Subjects

Patients with clinically diagnosed neurofibromatosis type 1 (Exp. 1a: *n* = 11, mean age 28.0 (range 17–44) years, Exp. 1b: *n* = 10, 27.8 years (range 19–44), Exp. 1c: *n* = 10, 27.8 years (range 19–44)) according to the criteria of the National Institutes of Health (NIH, 1988) participated in this study. Patients with NF1 and healthy controls were not treated with psychotropic medications. Inclusion criteria were age 18 to 45 years and right-handedness. Exclusion criteria were segmental NF1, deafness, severely impaired vision, use of central active drugs (e.g. methylphenidate). The control group in Exp. 1 consisted of age-matched healthy volunteers (Exp. 1a: *n* = 11, 24.73 ± 3.58 years, 5 female, 6 male (range 18–31 years) Exp. 1b: *n* = 10, 23.3 ± 3.77, 6 female, 4 male (range 19–30 years) aged; Exp. 1c: 22.0 ± 2.31, 6 female, 4 male (range 20–26 years). All subjects were self-reported right-handed and did not fulfil any exclusion criteria concerning the safety of TMS [33].

### Electromyographic recording

Subjects were seated comfortably in an armchair with their stimulated hand resting on a cushion. Motor-evoked potentials (MEPs) were recorded from the left abductor pollicis brevis (APB) muscle at rest by surface electromyography (EMG) using silver/silver chloride electrodes with a surface area of 263 mm^2^ (AMBU, Ballerup, Denmark) in belly-tendon recording technique. Data were band-pass filtered (20 – 2000 Hz) and amplified using an Ekida DC universal amplifier (EKIDA GmbH, Helmstadt, Germany), digitised at 5 kHz sampling rate using a MICRO1401*mk*II data acquisition unit (Cambridge Electronic Design Ltd, Cambridge, UK) and stored on a standard personal computer for online visual display and later offline analysis using Signal Software version 3 (CED Ltd, UK).

### Transcranial magnetic stimulation

For focal transcranial magnetic stimulation the intersection of a figure eight shaped stimulation coil with an external wing diameter of 90 mm was centred tangentially on the scalp over the motor cortex with its handle pointing in a posterior direction and laterally at an angle of approximately 45° away from the midline. Thus the current in the brain induced by magnetic stimulation was in a posterior-anterior direction, roughly perpendicular to the central sulcus and was therefore optimal for activating the neurons of the corticospinal pathways transsynaptically [34,35]. The coil was connected to a Magstim 200 Stimulator (The Magstim Company Ltd, Whitland, UK) which outputs a monophasic current waveform. By moving the coil over the motor cortex while administering stimuli of suprathreshold intensity, we could identify the optimal position for eliciting MEPs of maximum amplitudes from the target muscle (“hotspot”). The individual hotspot was recorded using an optically tracked navigation system (camera: NDI, Waterloo, Ontario, Canada; software: Fraunhofer Institute (IPA), Stuttgart, Germany) described previously [36] and thus kept constant throughout investigations. Magnetic stimuli were administered to the cortex at a frequency of 0.1 Hz, except stimuli used to identify the hotspot and motor threshold, which were administered at 0.25 Hz. We determined the resting motor threshold using a maximum-likelihood threshold-hunting procedure [37,38]. We used 16 TMS stimuli starting at 45% of maximum stimulator output. A positive MEP was defined as a muscle activation of >50 μV. The initial stimulator output for evaluation was chosen to target a mean MEP amplitude of 800–1200 μV (SI_1mV_) and was then kept constant throughout the investigations to assess changes in MEPs. MEP size was determined by measuring the two highest peaks of opposite polarity [29,39,40] and then averaged over 20 trials for each point of investigation. Subjects were asked to relax the target muscle during all measurements and relaxation was monitored by visual feedback via EMG-baseline.

### Paired associative stimulation (PAS)

Paired associative stimulation consisted of 200 pairs at a frequency of 0.25 Hz of peripheral electric stimulation of the left median nerve at the wrist, followed by TMS of the right M1 at the optimal site to elicit MEPs in the left APB muscle [41]. Electrical stimulation was applied through a Digitimer DS7 electrical stimulator (Digitimer Ltd, Welwyn Garden City, Hertfortshire, UK) using a bipolar electrode with the cathode proximal. We identified the optimal stimulation site at the wrist, affixed the electrode, and determined the threshold of perception. During PAS, constant current square wave pulses with duration of 1000 μs were applied at an intensity of three times the perceptual threshold. We employed the intensity for TMS which produced MEP amplitudes of, on average, 1 mV before the intervention (SI_1mV_). The same intensity was used for evaluation. The ISI between electrical and transcranial magnetic stimulation was 25 ms as this interval induces an increase in MEP amplitudes [19].

One crucial point when applying PAS is the subject’s attention level. It may influence the PAS effect’s magnitude. To avoid potential influence of attention on the results, we constantly reminded all the subjects to focus their attention on the stimulated hand and mentally count the number of electric stimuli. This assured a comparable level of attention between both groups (i.e. HC and NF1). Additionally, none of the patients reported of any diagnosed or relevant attention deficit in daily life.

As application of a high number of TMS stimuli might influence subsequent induction of plasticity [21], we limited the number of pre-interventional TMS stimuli in all experiments to 200. Each subject participated in studies of PAS where we measured MEP amplitudes and resting motor threshold before (PRE) as well as at three points in time after PAS (POST 1, POST 2 and POST 3). For experimental design see Figure 6.

### Short interval cortical inhibition

All experiments were performed by TMS with the target muscle at rest. To evoke SICI, a subthreshold conditioning stimulation was delivered 2, 3 and 5 ms before a test stimulus [23]. The stimulator output for test stimulus was adjusted to evoke mean MEP amplitude of 1 mV peak-to-peak.

The standard protocol for SICI has been optimized to maximize inhibitory effects of the preceding, conditioning stimulus using an intensity of 80% of resting motor threshold [42]. Using this paradigm increased inhibition in patients with NF1 compared to healthy subjects may not be detected because of a ceiling effect. Because it is known, that decreasing the conditioning stimulus intensity is lowering the inhibitory effect, we first created an intensity curve for SICI by using randomized conditioning stimulus intensities in trials of 0.5, 0.55, 0.6, 0.65, 0.7, 0.75 and 0.8 resting motor threshold [43,44]. Then we selected the stimulus intensity with lowest but still significant inhibition in the control group. This was the case using a conditioning stimulus intensity of 0.60 resting motor threshold (60%: 0.83 ± 0.42 to 0.58 ± 0.27, p = 0.017; 55%: 1.03 ± 0.51 to 1.0 ± 0.57, p = 0.575). This intensity was used to test the hypothesis (i) whether inhibition in patients with NF1 is increased and (ii) whether lovastatin leads to a disinhibition in patients with NF1.

With an interval of 4 s between trials, 10 conditioned MEPs were collected for each ISI, and in each experimental trial a total of 5 unconditioned test stimulus MEPs were recorded. Thus, for each conditioning pulse intensity 35 trials MEPs were recorded (3 ISIs with 10 MEPs each and 5 unconditioned MEPs). The order of data acquisition for each conditioning pulse intensity was randomized between subjects. The average of the amplitude of each conditioned MEP was expressed as a percentage of the average test stimulus MEP amplitude in the same session.

### Test for attentional performance (TAP)

Attention was measured through a computerized, standardized neuropsychological test, the TAP [45]. This test is widely used to evaluate attentional deficits including NF1. In order to be analogous to statin-responsive tests in animal studies of NF1, we choose four subtests of TAP: alertness and Go/No Go for basic attention and general anticipation; visual scanning for visual-spacial perceptivity (analogous to the morris water maze task for visual-spatial learning); incompatibility for divided visuospacial attention (analogous to the lateralized reaction time task).

### Alertness

In this test, reaction time is examined under two conditions. The first condition concerns simple reaction time measurements, in which a cross appears on the monitor a randomly varying intervals and to which the subjects should respond as quickly as possible by pressing a key. Intrinsic alertness is measured in this condition. In a second condition, reaction time is measured in response to a critical stimulus preceded by a cue stimulus presented as warning tone (WT). Thus, alertness is equivalent to anticipation and a degree of impulsiveness. It is the pre-requisite for effective behaviour, and is in this respect the basis of every attention performance.

### Visual scanning

In the visual scanning task, a matrix-like arrangement of 5 × 5 stimuli is used. Subjects have to “scan” this matrix and decide whether this arrangement includes a critical stimulus or not. One reaction key is used for the answer “present” and another for the answer “not present”. Thus, visual scanning tests the exploration of the special environment, which is one of the basic abilities subserving the safe movement in space.

### Go/No Go

In the go/no-go tasks, the subject has to react selectively to one class of stimuli but not to others. For testing a go/no-go-task was realized with 2 stimuli, squares with different textures, where two were targets. The aim of this examination is an assessment of the capacity of focused attention (reject irrelevant information). Thus, the go/no-go task tests the ability to suppress an inappropriate reaction, that is, control of impulsive behaviour.

### Incompatibility

In the incompatibility task, a right or left pointing arrow appears in the left or right side of the visual field. Here, the subjects have to press the button for the direction of the arrow but not the site where the arrow appears. This procedure tests the interference tendency in terms of stimulus-reaction incompatibility.

### Pharmacological intervention and randomization procedure

We performed the study in a placebo-controlled, randomized and double blind design for pharmacological interventions. Patients obtained 5 tablets containing 40 mg lovastatin (Mevacor, 1 A Pharma®) or placebo (lactose-monohydrate, magnesium stearate, cellulose powder). Patients and all other investigators were blind to the treatment allocation. Tablets were given every day at the same time at the day of examination, 2.8 h before PAS started. This was done because the time to reach peak plasma concentration is 2.8 hours [46]. Because this was a short term administration and the aim of this study was “proof of principle”, a dosage exceeding the clinical routine dosage was administered. It has been shown that doses of 200 mg of lovastatin were proven to be save in adult human volunteers [32], moreover overdose up to 5–6 g have been tolerated with no specific symptoms and recovery without sequelae. None of the participants reported any side effects.

### Data analysis

We ensured comparable relaxation in the recorded muscle while recording the TMS measurements visually. In addition, we investigated offline the EMG baseline of each trial. Pre-facilitated trials were excluded from further analyses. Trials that differed by over twice the standard deviation from the mean were left out of the analysis. We computed all statistical analyses using SPPS version 15.0 Software (SPSS Inc., Chicago, IL, USA). Before any further analysis, data were tested for normality. For experiments 1, statistical evaluation was performed via a repeated-measures analysis of variance (rmANOVA) with group (two levels: CONTROL and NF1) as between subject factor and time (four levels: PRE, POST 1, POST 2 and POST 3) as within-subjects factor. For experiment 2, we performed rmANOVA with drug (two levels: ON and OFF) and time (four levels: PRE, POST 1, POST 2 and POST 3) as within-subject factors. We used the Greenhouse-Geisser correction to adjust for violations of sphericity, if necessary. In case of significant main effects, we employed post-hoc two-tailed Student’s paired or unpaired *t*-tests without correction. This method was used for MEP and resting motor threshold values. To analyse baseline parameters, we used two-tailed Student’s paired *t* tests. We compared baseline MEP amplitudes using a one-way analysis of variance (ANOVA) with experiment as factor. MEP amplitudes of each subject in experiment 2 were analysed using an rmANOVA with TIME (four levels: PRE, POST 1, POST 2 and POST 3) as within-subject factor. The significance level was set at 0.05 in all analyses. All values given are mean group values ± standard deviation, if not indicated otherwise.

## Abbreviations

LTP: Long term potentiation; RAS: Rat sarcoma; HC: Healty controls; NF1: Neurofibromatosis type 1; TMS: Transcranial magnetic stimulation; TAP: Testbattery for attentional performance; GABA: Gamma aminobutyric acid; MAPK: Mitogen-activated protein kinase; SICI: Short intracortical inhibition; PAS: Paired associated inhibition; MEP: Motor evoked potential.

## Competing interests

The authors declare that they have no competing interests.

## Authors’ contributions

FM drafted the manuscript, had substantial contributions to design of the study and data acquisition, NHJ drafted parts of the manuscript, had substantial contributions to study design and acquisition of the data, MZ and UW have been involved in study design, recruitment of participants and genetically analyses and revised the manuscript critically. LF, SL, SG had substantial contributions to data acquisition and revised the manuscript critically. SB, TW, AS, FH, VFM and KL had substantial contributions in data acquisition and revised the manuscript critically and approved the final version. VM had substantial contributions to study design and conception, drafted the manuscript and approved the final version. All authors listed had complete access to the data and have given final approval of the version to be published. Please see sample text in the instructions for authors.

## Pre-publication history

The pre-publication history for this paper can be accessed here:

http://www.biomedcentral.com/1471-2377/13/131/prepub
